# 20-Oxa­penta­cyclo­[15.2.1.0^2,16^.0^3,8^.0^10,15^]icosa-2(16),3,5,7,10(15),11,13,18-octaen-9-one

**DOI:** 10.1107/S2414314626004992

**Published:** 2026-05-15

**Authors:** Dieter Schollmeyer, Heiner Detert

**Affiliations:** a University of Mainz, Department of Chemistry, Duesbergweg 10-14, 55099 Mainz, Germany; Goethe-Universität Frankfurt, Germany

**Keywords:** crystal structure, tropone, cyclo­alkyne

## Abstract

The mol­ecules of the title compound, C_19_H_12_O_2_, adopt a saddle shape, the *o*-xylylene fragments are almost planar. The carbonyl group and the ether oxygen atom are located on the same side of the carbon skeleton. The mol­ecules are arranged in strands with alternating directions.

## Abstract 2

The mol­ecules of the title compound, C_19_H_12_O_2_, adopt a saddle shape with almost planar *o*-xylylene fragments. The carbonyl group and the ether oxygen atom are located on the same side of the carbon skeleton. The mol­ecules are arranged in strands with alternating directions.

## Graphical abstract

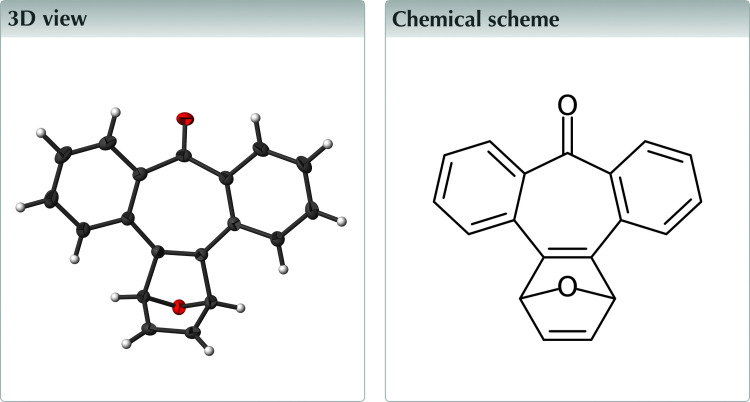

## Structure description

Our long-standing inter­est in the chemistry of strained cyclo­alkynes of medium ring size (Detert & Meier, 1997 ▸; Detert *et al.*, 1994 ▸) and even seven-membered rings (Herges *et al.*, 2005 ▸; Schollmeyer & Detert, 2023 ▸) led to the reinvestigation of dibenzo­cyclo­heptynone. This compound was trapped in a Diels–Alder reaction with furane (Tochtermann *et al.*, 1964*a* ▸). This adduct of dibenzo­cyclo­heptynone is a starting material for the synthesis of dibenzofurotropone (Sasaki *et al.*, 1976 ▸).

**Figure 1:**
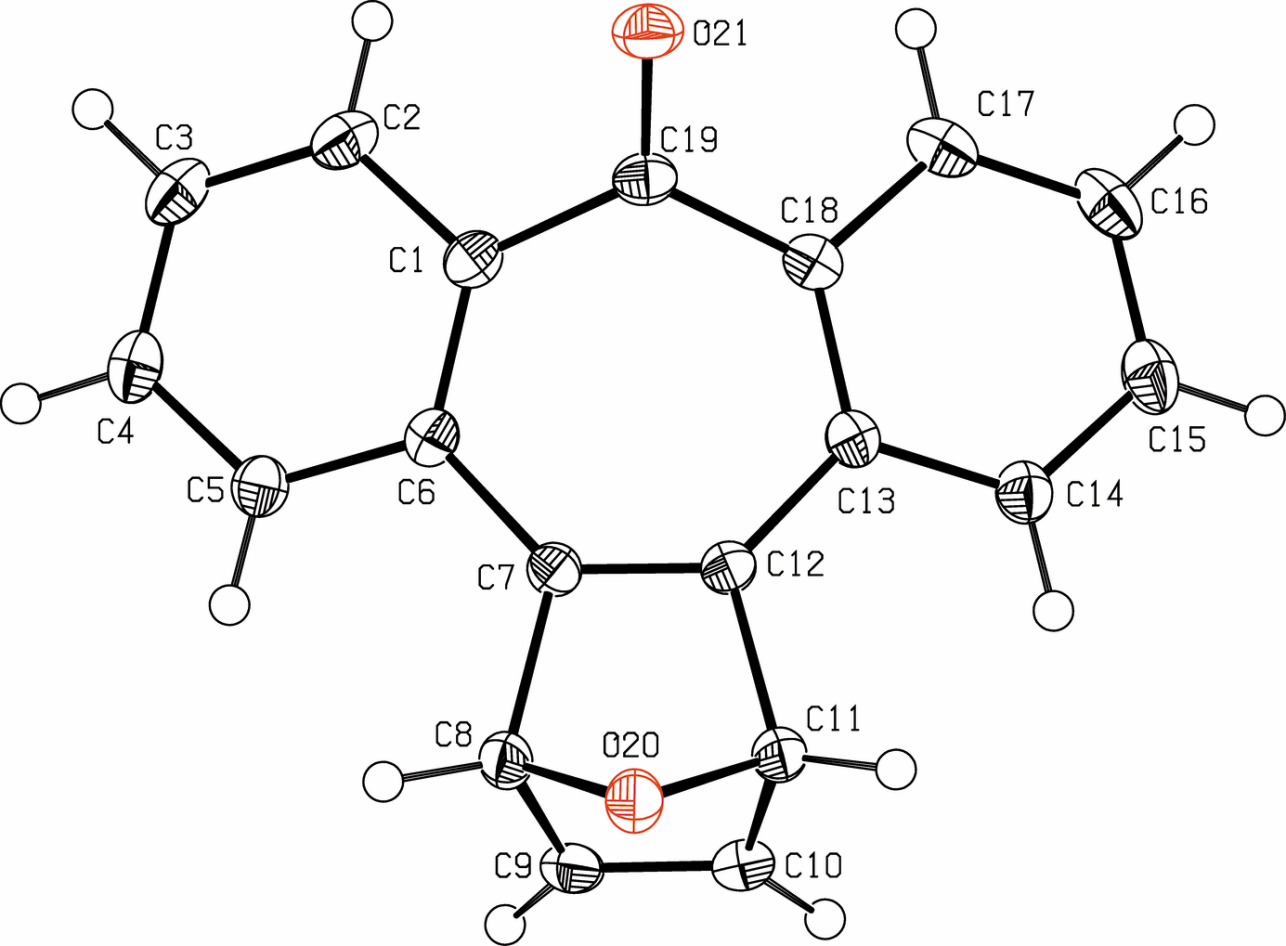
View of the title compound. Displacement ellipsoids are drawn at the 50% probability level.

The title mol­ecule is shown in Fig. 1 ▸. Four mol­ecules fill the monoclinic unit cell. The mol­ecules are arranged in strands parallel to the *c* axis. Translational symmetry connects the mol­ecules within a strand while a center of inversion connects mol­ecules in parallel strands. As the strands are quite close, the phenyl ring C13–C18 lies over the tropone ring of the next mol­ecule in the vicinal strand. The mol­ecular shape of this virtually mirror symmetrical penta­cyclic compound is that of a saddle, the carbonyl group being the pommel, the bicyclic ether group the cantle and the phenyl rings the flaps. The latter subtend a dihedral angle of 28.04 (5)°. The *ortho*-disubstituted phenyl rings are almost planar: significant deviations from mean plane of C1–C6 are −0.028 (2) Å at C7 and −0.023 (2) Å at C19; deviations from mean plane of C13–C18 are −0.024 (2) Å at C12 and +0.142 (2) Å at C19. The atoms C1, C6, C7, C12, C13, C18 of the tropone moiety are almost coplanar, with a maximum deviation of 0.1317 (9) Å at C16. Atom C19 lies 0.4876 (16) Å above this plane and therefore, the carbonyl group peaks out of the mol­ecular plane. The carbonyl and the ether oxygen atoms are on the same side of the mol­ecule.

**Figure 2:**
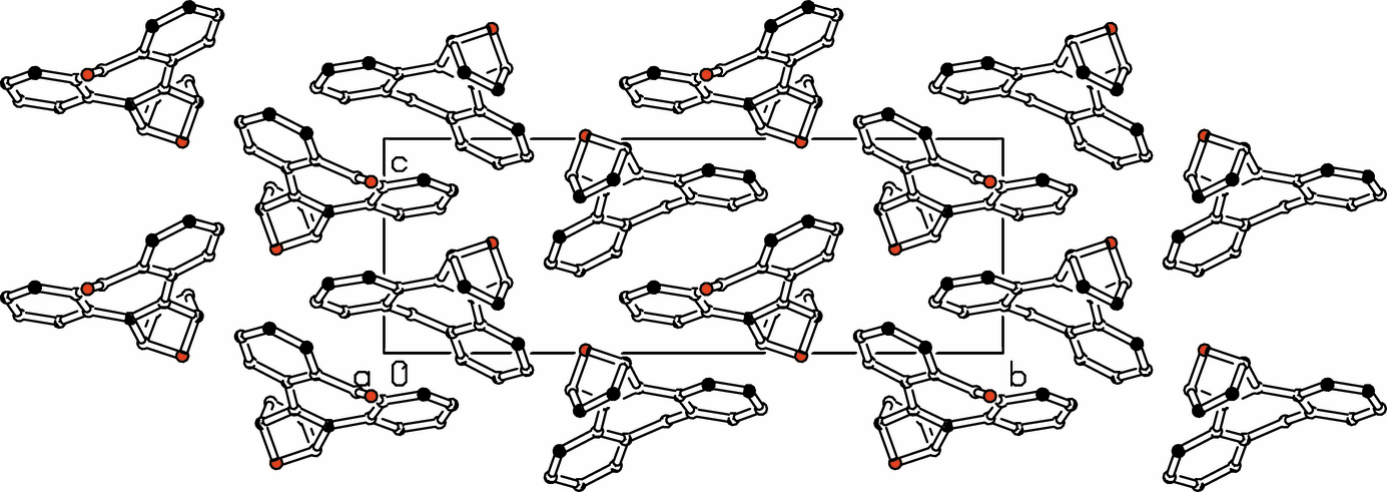
Part of the packing diagram. View along *a*-axis direction. Hydrogen atoms removed for clarity.

In the extended structure, the mol­ecules are linked by C—H⋯O hydrogen bonds (Fig. 2 ▸, Table 1 ▸).

## Synthesis and crystallization

The title compound was prepared by de­hydro­bromination of 4-bromo­[2.3;6.7]-dibenzo­cyclo­hepta­trienone with potassium *tert*-butyl­ate in the presence of furane according to Tochtermann (Tochtermann *et al.*, 1964*a* ▸,*b* ▸). The compound was obtained in 67% yield as slightly beige crystals, m.p. = 477–479 K. ^1^H-NMR (CDCl_3_, 400 MHz;): 6.04 (*s*, 2 H, OCH, *J*_CH_ = 160 Hz). 7.39 (*dd*, 2 H, *J ca* 1.6 Hz, olefin), 7.49 (*dd*, 2 H, *J* = 8 Hz, *J*′= 1.5 Hz), 7.56 (*ddd*, 2 H, *J* = 7.8 Hz, *J*′= 1.8 Hz), 7.69 (*ddd*, 2 H, *J* = *J*′= 8 Hz, *J*′′= 1.3 Hz), 8.10 (*dd*, 2 H, *J* = 8 Hz, *J*′= 1.3 Hz). ^13^C-NMR (CDCl_3_, 100 MHz; #*x.yz* = HMBC cross coupling to H-NMR signal): 85.51, (O—CH, #6.04), 123,43 (CH, #7.49), 129.00 (CH, #7.56), 129.75 (CH, #8.10), 131.69 (Cq), 131.88 (CH, #7.68), 137.97 (Cq), 142.14 (CH, olefin, #7.39), 148.81 (Cq), 194.02 (C=O).

## Refinement

Crystal data, data collection and structure refinement details are summarized in Table 2 ▸.

## Supplementary Material

Crystal structure: contains datablock(s) I, global. DOI: 10.1107/S2414314626004992/bt4199sup1.cif

Structure factors: contains datablock(s) I. DOI: 10.1107/S2414314626004992/bt4199Isup2.hkl

Supporting information file. DOI: 10.1107/S2414314626004992/bt4199Isup3.cml

CCDC reference: 2553510

Additional supporting information:  crystallographic information; 3D view; checkCIF report

## Figures and Tables

**Table 1:** Hydrogen-bond geometry (Å, °)

*D*—H⋯*A*	*D*—H	H⋯*A*	*D*⋯*A*	*D*—H⋯*A*
C3—H3⋯O20^i^	0.95	2.51	3.3886 (17)	153
C10—H10⋯O21^ii^	0.95	2.48	3.3963 (18)	162

Symmetry codes: (i) 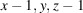; (ii) 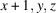.

**Table 2:** Experimental details

Crystal data	
Chemical formula	C_19_H_12_O_2_
*M*_r_	272.29
Crystal system, space group	Monoclinic, *P*2_1_/*c*
Temperature (K)	120
*a*, *b*, *c* (Å)	8.5654 (3), 20.7138 (6), 7.4518 (3)
β (°)	105.809 (3)
*V* (Å^3^)	1272.10 (8)
*Z*	4
Radiation type	Mo *K*α
μ (mm^−1^)	0.09
Crystal size (mm)	0.55 × 0.35 × 0.17

Data collection	
Diffractometer	Stoe *IPDS* 2T
No. of measured, independent and observed [*I* > 2σ(*I*)] reflections	8154, 3014, 2654
*R*_int_	0.020
(sin θ/λ)_max_ (Å^−1^)	0.658

Refinement	
*R*[*F*^2^ > 2σ(*F*^2^)], *wR*(*F*^2^), *S*	0.042, 0.114, 1.02
No. of reflections	3014
No. of parameters	190
H-atom treatment	H-atom parameters constrained
Δρ_max_, Δρ_min_ (e Å^−3^)	0.35, −0.20

Computer programs: *X-AREA WinXpose* (Stoe & Cie, 2020*a* ▸), *X-AREA Recipe* (Stoe & Cie, 2020*b* ▸), *X-AREA Integrate* (Stoe & Cie, 2020*c* ▸), *SHELXT2014* (Sheldrick, 2015*a* ▸), *SHELXL2019/2* (Sheldrick, 2015*b* ▸) and *PLATON* (Spek, 2009 ▸).

## full crystallographic data

### 20-Oxapentacyclo[15.2.1.02,16.03,8.010,15]icosa-2(16),3,5,7,10(15),11,13,18-octaen-9-one Crystal data

**Table:**

C_19_H_12_O_2_	*F*(000) = 568
*M**_r_* = 272.29	*D*_x_ = 1.422 Mg m^−^^3^
Monoclinic, *P*2_1_/*c*	Mo *K*α radiation, λ = 0.71073 Å
*a* = 8.5654 (3) Å	Cell parameters from 12059 reflections
*b* = 20.7138 (6) Å	θ = 2.7–28.4°
*c* = 7.4518 (3) Å	µ = 0.09 mm^−^^1^
β = 105.809 (3)°	*T* = 120 K
*V* = 1272.10 (8) Å^3^	Block, colorless
*Z* = 4	0.55 × 0.35 × 0.17 mm

### 20-Oxapentacyclo[15.2.1.02,16.03,8.010,15]icosa-2(16),3,5,7,10(15),11,13,18-octaen-9-one Data collection

**Table:**

Stoe IPDS 2T diffractometer	*R*_int_ = 0.020
Detector resolution: 6.67 pixels mm^-1^	θ_max_ = 27.9°, θ_min_ = 2.7°
rotation method, ω scans	*h* = −11→11
8154 measured reflections	*k* = −27→27
3014 independent reflections	*l* = −9→7
2654 reflections with *I* > 2σ(*I*)	

### 20-Oxapentacyclo[15.2.1.02,16.03,8.010,15]icosa-2(16),3,5,7,10(15),11,13,18-octaen-9-one Refinement

**Table:**

Refinement on *F*^2^	Primary atom site location: dual
Least-squares matrix: full	Hydrogen site location: inferred from neighbouring sites
*R*[*F*^2^ > 2σ(*F*^2^)] = 0.042	H-atom parameters constrained
*wR*(*F*^2^) = 0.114	*w* = 1/[σ^2^(*F*_o_^2^) + (0.0557*P*)^2^ + 0.7054*P*] where *P* = (*F*_o_^2^ + 2*F*_c_^2^)/3
*S* = 1.02	(Δ/σ)_max_ < 0.001
3014 reflections	Δρ_max_ = 0.35 e Å^−^^3^
190 parameters	Δρ_min_ = −0.20 e Å^−^^3^
0 restraints	

### 20-Oxapentacyclo[15.2.1.02,16.03,8.010,15]icosa-2(16),3,5,7,10(15),11,13,18-octaen-9-one Special details

**Table:**

Geometry. All esds (except the esd in the dihedral angle between two l.s. planes) are estimated using the full covariance matrix. The cell esds are taken into account individually in the estimation of esds in distances, angles and torsion angles; correlations between esds in cell parameters are only used when they are defined by crystal symmetry. An approximate (isotropic) treatment of cell esds is used for estimating esds involving l.s. planes.
Refinement. Hydrogen atoms were placed at calculated positions and were refined in the riding-model approximation with C_tertiary_–H = 1.00 Å or with C–H = 0.95 Å for the remaining H atoms. *U*_iso_(H) were set to 1.2 *U*_eq_(C).

### 20-Oxapentacyclo[15.2.1.02,16.03,8.010,15]icosa-2(16),3,5,7,10(15),11,13,18-octaen-9-one Fractional atomic coordinates and isotropic or equivalent isotropic displacement parameters (Å^2^)

**Table:**

	*x*	*y*	*z*	*U*_iso_*/*U*_eq_	
C1	0.25685 (15)	0.39001 (6)	0.59801 (17)	0.0202 (3)	
C2	0.10665 (16)	0.37416 (7)	0.47387 (19)	0.0251 (3)	
H2	0.021235	0.404937	0.449857	0.030*	
C3	0.07999 (17)	0.31493 (7)	0.3858 (2)	0.0271 (3)	
H3	−0.022313	0.305356	0.301377	0.033*	
C4	0.20426 (17)	0.26925 (7)	0.42163 (19)	0.0251 (3)	
H4	0.186619	0.228211	0.362647	0.030*	
C5	0.35289 (16)	0.28386 (6)	0.54301 (18)	0.0214 (3)	
H5	0.436610	0.252354	0.566258	0.026*	
C6	0.38439 (15)	0.34424 (6)	0.63376 (17)	0.0185 (3)	
C7	0.54171 (15)	0.35550 (6)	0.76243 (17)	0.0183 (3)	
C8	0.67373 (16)	0.30275 (6)	0.82756 (19)	0.0222 (3)	
H8	0.633420	0.257237	0.818979	0.027*	
C9	0.80783 (16)	0.31660 (7)	0.7309 (2)	0.0243 (3)	
H9	0.833944	0.291985	0.635415	0.029*	
C10	0.87911 (16)	0.37041 (7)	0.80974 (19)	0.0239 (3)	
H10	0.967473	0.392565	0.783467	0.029*	
C11	0.78726 (15)	0.38891 (6)	0.95271 (18)	0.0209 (3)	
H11	0.845070	0.419093	1.053782	0.025*	
C12	0.61587 (15)	0.41033 (6)	0.83825 (17)	0.0181 (3)	
C13	0.57029 (15)	0.47724 (6)	0.80355 (17)	0.0182 (3)	
C14	0.69311 (16)	0.52443 (6)	0.85300 (19)	0.0224 (3)	
H14	0.801901	0.511295	0.908102	0.027*	
C15	0.65888 (18)	0.58935 (7)	0.8231 (2)	0.0269 (3)	
H15	0.743349	0.620381	0.858885	0.032*	
C16	0.50090 (19)	0.60910 (7)	0.7409 (2)	0.0281 (3)	
H16	0.477393	0.653629	0.717555	0.034*	
C17	0.37795 (18)	0.56411 (7)	0.6929 (2)	0.0252 (3)	
H17	0.269925	0.578152	0.637738	0.030*	
C18	0.40932 (15)	0.49792 (6)	0.72410 (17)	0.0194 (3)	
C19	0.26232 (15)	0.45601 (6)	0.68114 (17)	0.0204 (3)	
O20	0.74991 (12)	0.32597 (4)	1.01305 (13)	0.0243 (2)	
O21	0.13591 (12)	0.47904 (5)	0.70074 (15)	0.0276 (2)	

### 20-Oxapentacyclo[15.2.1.02,16.03,8.010,15]icosa-2(16),3,5,7,10(15),11,13,18-octaen-9-one Atomic displacement parameters (Å^2^)

**Table:**

	*U*^11^	*U*^22^	*U*^33^	*U*^12^	*U*^13^	*U*^23^
C1	0.0187 (6)	0.0236 (6)	0.0178 (6)	−0.0009 (5)	0.0045 (5)	0.0008 (5)
C2	0.0182 (6)	0.0329 (7)	0.0226 (6)	0.0004 (5)	0.0029 (5)	−0.0011 (5)
C3	0.0197 (6)	0.0361 (8)	0.0232 (6)	−0.0056 (5)	0.0018 (5)	−0.0034 (5)
C4	0.0266 (7)	0.0264 (7)	0.0219 (6)	−0.0076 (5)	0.0061 (5)	−0.0045 (5)
C5	0.0219 (6)	0.0214 (6)	0.0212 (6)	−0.0018 (5)	0.0064 (5)	0.0001 (5)
C6	0.0188 (6)	0.0200 (6)	0.0166 (5)	−0.0023 (5)	0.0046 (5)	0.0014 (4)
C7	0.0190 (6)	0.0170 (6)	0.0183 (6)	0.0005 (4)	0.0043 (5)	0.0024 (4)
C8	0.0223 (6)	0.0170 (6)	0.0233 (6)	0.0003 (5)	−0.0005 (5)	0.0011 (5)
C9	0.0199 (6)	0.0229 (6)	0.0275 (7)	0.0062 (5)	0.0019 (5)	−0.0017 (5)
C10	0.0169 (6)	0.0247 (6)	0.0285 (7)	0.0028 (5)	0.0032 (5)	−0.0008 (5)
C11	0.0195 (6)	0.0188 (6)	0.0220 (6)	0.0004 (5)	0.0013 (5)	0.0003 (5)
C12	0.0173 (6)	0.0193 (6)	0.0174 (6)	0.0003 (4)	0.0042 (4)	0.0005 (4)
C13	0.0210 (6)	0.0184 (6)	0.0162 (6)	−0.0001 (5)	0.0067 (5)	−0.0008 (4)
C14	0.0236 (6)	0.0207 (6)	0.0238 (6)	−0.0019 (5)	0.0079 (5)	−0.0012 (5)
C15	0.0342 (7)	0.0195 (6)	0.0310 (7)	−0.0053 (5)	0.0158 (6)	−0.0020 (5)
C16	0.0400 (8)	0.0166 (6)	0.0327 (7)	0.0038 (5)	0.0185 (6)	0.0029 (5)
C17	0.0296 (7)	0.0224 (7)	0.0260 (7)	0.0071 (5)	0.0115 (5)	0.0044 (5)
C18	0.0219 (6)	0.0196 (6)	0.0174 (6)	0.0023 (5)	0.0067 (5)	0.0011 (5)
C19	0.0188 (6)	0.0242 (6)	0.0172 (6)	0.0035 (5)	0.0033 (5)	0.0028 (5)
O20	0.0263 (5)	0.0200 (5)	0.0219 (5)	−0.0009 (4)	−0.0013 (4)	0.0030 (4)
O21	0.0206 (5)	0.0312 (5)	0.0308 (5)	0.0054 (4)	0.0068 (4)	−0.0021 (4)

### 20-Oxapentacyclo[15.2.1.02,16.03,8.010,15]icosa-2(16),3,5,7,10(15),11,13,18-octaen-9-one Geometric parameters (Å, º)

**Table:**

C1—C2	1.4036 (18)	C10—C11	1.5355 (19)
C1—C6	1.4159 (17)	C10—H10	0.9500
C1—C19	1.4963 (18)	C11—O20	1.4432 (15)
C2—C3	1.381 (2)	C11—C12	1.5483 (17)
C2—H2	0.9500	C11—H11	1.0000
C3—C4	1.394 (2)	C12—C13	1.4440 (17)
C3—H3	0.9500	C13—C14	1.4096 (18)
C4—C5	1.3796 (19)	C13—C18	1.4107 (17)
C4—H4	0.9500	C14—C15	1.3814 (19)
C5—C6	1.4123 (18)	C14—H14	0.9500
C5—H5	0.9500	C15—C16	1.386 (2)
C6—C7	1.4441 (17)	C15—H15	0.9500
C7—C12	1.3480 (17)	C16—C17	1.378 (2)
C7—C8	1.5522 (17)	C16—H16	0.9500
C8—O20	1.4409 (16)	C17—C18	1.4042 (18)
C8—C9	1.540 (2)	C17—H17	0.9500
C8—H8	1.0000	C18—C19	1.4906 (18)
C9—C10	1.3285 (19)	C19—O21	1.2278 (16)
C9—H9	0.9500		
			
C2—C1—C6	119.28 (12)	C11—C10—H10	127.6
C2—C1—C19	113.97 (11)	O20—C11—C10	100.93 (10)
C6—C1—C19	126.75 (11)	O20—C11—C12	99.97 (10)
C3—C2—C1	121.62 (13)	C10—C11—C12	106.10 (10)
C3—C2—H2	119.2	O20—C11—H11	115.9
C1—C2—H2	119.2	C10—C11—H11	115.9
C2—C3—C4	119.54 (12)	C12—C11—H11	115.9
C2—C3—H3	120.2	C7—C12—C13	131.30 (12)
C4—C3—H3	120.2	C7—C12—C11	104.91 (11)
C5—C4—C3	119.78 (13)	C13—C12—C11	122.90 (11)
C5—C4—H4	120.1	C14—C13—C18	118.27 (12)
C3—C4—H4	120.1	C14—C13—C12	118.21 (11)
C4—C5—C6	122.01 (13)	C18—C13—C12	123.52 (11)
C4—C5—H5	119.0	C15—C14—C13	121.50 (13)
C6—C5—H5	119.0	C15—C14—H14	119.3
C5—C6—C1	117.76 (11)	C13—C14—H14	119.3
C5—C6—C7	118.88 (11)	C14—C15—C16	119.81 (13)
C1—C6—C7	123.32 (11)	C14—C15—H15	120.1
C12—C7—C6	131.48 (12)	C16—C15—H15	120.1
C12—C7—C8	104.01 (11)	C17—C16—C15	119.99 (13)
C6—C7—C8	124.22 (11)	C17—C16—H16	120.0
O20—C8—C9	100.72 (10)	C15—C16—H16	120.0
O20—C8—C7	99.55 (10)	C16—C17—C18	121.26 (13)
C9—C8—C7	107.17 (10)	C16—C17—H17	119.4
O20—C8—H8	115.7	C18—C17—H17	119.4
C9—C8—H8	115.7	C17—C18—C13	119.14 (12)
C7—C8—H8	115.7	C17—C18—C19	114.88 (12)
C10—C9—C8	105.11 (12)	C13—C18—C19	125.80 (11)
C10—C9—H9	127.4	O21—C19—C18	117.95 (12)
C8—C9—H9	127.4	O21—C19—C1	118.36 (12)
C9—C10—C11	104.82 (12)	C18—C19—C1	123.40 (11)
C9—C10—H10	127.6	C8—O20—C11	94.80 (9)
			
C6—C1—C2—C3	0.1 (2)	O20—C11—C12—C13	156.23 (11)
C19—C1—C2—C3	−179.44 (12)	C10—C11—C12—C13	−99.23 (13)
C1—C2—C3—C4	0.6 (2)	C7—C12—C13—C14	−158.24 (14)
C2—C3—C4—C5	−0.7 (2)	C11—C12—C13—C14	9.31 (18)
C3—C4—C5—C6	0.0 (2)	C7—C12—C13—C18	22.2 (2)
C4—C5—C6—C1	0.66 (19)	C11—C12—C13—C18	−170.22 (12)
C4—C5—C6—C7	178.52 (12)	C18—C13—C14—C15	−0.79 (19)
C2—C1—C6—C5	−0.71 (18)	C12—C13—C14—C15	179.66 (12)
C19—C1—C6—C5	178.77 (12)	C13—C14—C15—C16	−0.7 (2)
C2—C1—C6—C7	−178.47 (12)	C14—C15—C16—C17	1.4 (2)
C19—C1—C6—C7	1.0 (2)	C15—C16—C17—C18	−0.6 (2)
C5—C6—C7—C12	164.97 (13)	C16—C17—C18—C13	−0.9 (2)
C1—C6—C7—C12	−17.3 (2)	C16—C17—C18—C19	174.62 (12)
C5—C6—C7—C8	−7.79 (18)	C14—C13—C18—C17	1.60 (18)
C1—C6—C7—C8	169.95 (12)	C12—C13—C18—C17	−178.87 (12)
C12—C7—C8—O20	37.21 (12)	C14—C13—C18—C19	−173.43 (12)
C6—C7—C8—O20	−148.37 (11)	C12—C13—C18—C19	6.10 (19)
C12—C7—C8—C9	−67.23 (13)	C17—C18—C19—O21	−30.25 (17)
C6—C7—C8—C9	107.18 (13)	C13—C18—C19—O21	144.96 (13)
O20—C8—C9—C10	−34.00 (13)	C17—C18—C19—C1	143.51 (12)
C7—C8—C9—C10	69.61 (13)	C13—C18—C19—C1	−41.28 (19)
C8—C9—C10—C11	−0.28 (13)	C2—C1—C19—O21	28.98 (17)
C9—C10—C11—O20	34.44 (13)	C6—C1—C19—O21	−150.53 (13)
C9—C10—C11—C12	−69.40 (13)	C2—C1—C19—C18	−144.76 (12)
C6—C7—C12—C13	−6.8 (2)	C6—C1—C19—C18	35.74 (19)
C8—C7—C12—C13	167.04 (13)	C9—C8—O20—C11	53.20 (11)
C6—C7—C12—C11	−175.99 (13)	C7—C8—O20—C11	−56.47 (10)
C8—C7—C12—C11	−2.16 (13)	C10—C11—O20—C8	−53.57 (11)
O20—C11—C12—C7	−33.43 (12)	C12—C11—O20—C8	55.14 (11)
C10—C11—C12—C7	71.12 (12)		

### 20-Oxapentacyclo[15.2.1.02,16.03,8.010,15]icosa-2(16),3,5,7,10(15),11,13,18-octaen-9-one Hydrogen-bond geometry (Å, º)

**Table:**

*D*—H···*A*	*D*—H	H···*A*	*D*···*A*	*D*—H···*A*
C3—H3···O20^i^	0.95	2.51	3.3886 (17)	153
C10—H10···O21^ii^	0.95	2.48	3.3963 (18)	162

Symmetry codes: (i) *x*−1, *y*, *z*−1; (ii) *x*+1, *y*, *z*.
